# A Review of Hepatorenal Syndrome

**DOI:** 10.7759/cureus.16084

**Published:** 2021-07-01

**Authors:** Abinash Subedi, Vishnu Charan Suresh Kumar, Aakritee Sharma Subedi, Bishnu Sapkota

**Affiliations:** 1 Internal Medicine, State University of New York (SUNY) Upstate Medical University, Syracuse, USA; 2 Medicine, Chitwan Medical College Teaching Hospital, Bharatpur, NPL; 3 Gastroenterology, State University of New York (SUNY) Upstate Medical University, Syracuse, USA; 4 Gastroenterology, Veterans Affairs (VA) Medical Center, Syracuse, USA

**Keywords:** hepatorenal syndrome, octreotide, liver transplant, terlipressin, midodrine

## Abstract

The development of acute kidney injury (AKI) is one of the most frequent complications in patients with cirrhosis. AKI due to volume depletion is the most common etiology and hepatorenal syndrome (HRS) is the second most common cause of AKI in these patients. HRS is the extreme form of kidney injury in patients with cirrhosis, which is caused due to a reduction in renal blood flow unresponsive to volume expansion.* *The literature involving HRS is rapidly evolving and newer tests and updated definitions have been proposed which allows timely identification and treatment. Here, we will discuss the definition, pathophysiology, prevention, diagnosis, and treatment of HRS.

## Introduction and background

Hepatorenal syndrome (HRS) is one of the most common and severe complications of cirrhosis, which occurs due to functional reduction in circulatory volume to kidneys which is unresponsive to volume expansion. Historically, two subtypes of HRS are described. HRS type 1 is acute in onset (less than two weeks) and heralds a poor prognosis. HRS type 2 is more chronic in nature and is associated with less severe kidney dysfunction characterized by refractory ascites resistant to diuretics. The literature and understanding of the pathophysiology regarding HRS is rapidly evolving and we now have a more detailed understanding of the disease. Over the years, the definition of HRS has evolved and now allows for early diagnosis and treatment with subsequent improvement in overall outcomes. Diagnosis of HRS requires exclusion of other causes of kidney injury, including intrinsic causes such as acute tubular necrosis and postrenal causes. Management includes treatment with vasoconstrictors and albumin. Liver transplantation is the only curative treatment of HRS.

## Review

Definition of HRS

HRS is one of the severe complications occurring in patients with advanced cirrhosis, with high mortality if not recognized early and treated promptly. [1] It occurs due to the decrease in renal blood flow and subsequent reduction in glomerular filtration rate (GFR) unresponsive to volume expansion.

Acute renal impairment occurs in about 27%-53% of patients admitted to the hospital with decompensated cirrhosis and ascites [2]. Acute renal failure in cirrhosis was originally described by the International Club of Ascites in 1990 as an increase in serum creatinine of at least 50% from baseline value to ≥1.5 mg/dl. In 2007, ICA updated the definition and classified HRS into two different subtypes: Type 1 HRS and type 2 HRS. Type 1 HRS is characterized by abrupt deterioration of renal function by doubling of serum creatinine to a concentration of at least 2.5 mg/dl or a 50% reduction in the initial 24-hour creatinine clearance to below 20 ml/minute in less than two weeks. Type 2 HRS is a more gradual impairment in renal function that is less severe than type 1 HRS and has refractory ascites, which are resistant to diuretics [3].

However, serum creatinine is not a reliable predictor and often underestimates the degree of kidney damage in cirrhotic patients. Malnutrition and sarcopenia with reduced hepatic creatinine production, increased renal tubular secretion of creatinine, increased volume of distribution due to volume overload, and inaccurate measurement of serum creatinine by calorimetric method due to elevated serum bilirubin often leads to falsely lower serum creatinine level [4]. In practice, serum creatinine continues to be used for estimation of GFR, monitoring renal function, and diagnosing AKI and HRS. This is because the test is simple, broadly available, and inexpensive. 

In patients with cirrhosis, a rise in creatinine (by 0.3 mg/dl or 50% from baseline) is associated with significant morbidity and economic loss due to a longer hospital stay, admission to intensive care unit, multiple organ failure, and 90-day hospital mortality [5-7]. The reversal of HRS is inversely associated with the degree of creatinine elevation at the start of treatment, and the two weeks doubling time required to diagnose HRS type 1 in the older nomenclature could potentially lead to a delay in starting specific treatment for HRS [8,9]. The ICA has therefore updated the previous definitions and classifications of AKI in patients with cirrhosis in 2015 which align with the Kidney Disease Improving Global Outcomes (KIDGO) classification. The old term HRS type 1 has been replaced with HRS-AKI. Functional kidney injury in cirrhotic patients who do not meet the criteria of HRS-AKI is termed HRS-NAKI (HRS-Non-AKI) and is based on estimated glomerular filtration rate (eGFR) rather than the serum creatinine [10]. HRS-NAKI is further divided into two subtypes; HRS - Acute Kidney Disease (HRS-AKD) and HRS-CKD. [11] The new diagnostic criteria and classification of AKI according to ICA are elaborated in Table 1 and Table 2.

**Table 1:** New diagnostic criteria for HRS-AKI. *The evaluation of this parameter requires a urinary catheter. **This criterion would not be included in cases of known pre-existing structural chronic kidney disease (e.g., diabetic or hypertensive nephropathy). AKI: acute kidney injury; FENa: fractional excretion of sodium; HRS: hepatorenal syndrome; ICA: International Club of Ascites.

Cirrhosis, acute liver failure, or acute-on-chronic liver failure
Increase in serum creatinine ≥ 0.3 mg/dl within 48 hours or ≥ 50% from baseline value according to ICA consensus document and or urine ≤ 0.5 ml/kg bodyweight for ≥ 6 hours*
No full or partial response after at least two days of diuretic withdrawal and volume expansion with albumin. The recommended dose of albumin is 1 g/kg of body weight per day to a maximum of 100 g/day
Absence of shock
Absence of parenchymal disease as indicated by proteinuria >500 mg/day, microhematuria (>50 red blood cells per high power field), urinary injury biomarkers (if available), and or abnormal renal ultrasonography**
Suggestion of renal vasoconstriction with FENa of < 0.2% (with levels <0.1% being highly predictive)

**Table 2:** Old and the new classification of HRS. *Fulfillment of all the new ICA criteria for the diagnosis of HRS, *the evaluation of this parameter requires a urinary catheter. ICA: International Club of Ascites; AKI: acute kidney injury; AKD: acute kidney disease; CKD: chronic kidney disease; HRS: hepatorenal syndrome; SCr: serum creatinine; NAKI: non-acute kidney injury.

Old classification	New classification	Criteria
HRS type 1	HRS-AKI	Absolute increase in SCr ≥ 0.3mg/dl within 48 hours and/or
		Urine output ≤ 0.5 ml/kg body weight for ≥ 6 hours* or
		Percent increase in SCr ≥ 50% using the last available value of outpatient SCr within 3 months as the baseline value
HRS type 2	HRS-NAKI	
	HRS-AKD	eGFR < 60 ml/min per 1.73 m^2^ for < 3 months in absence of other (structural) causes
		Percent increase in SCr < 50% using the last available value of outpatient SCr within three months as the baseline value
	HRS-CKD	eGFR < 60 ml/min per 1.73 m^2^ for ≥ 3 months in absence of other (structural) causes

Pathophysiology

The main hypothesis regarding the development of HRS in the last 20 years has been the “splanchnic arterial vasodilation theory”. Cirrhosis is characterized by reduced systemic vascular resistance due to splanchnic vasodilation [12]. In the early stages of cirrhosis, a compensatory increase in cardiac output maintains the circulatory volume. However, the patient is susceptible to decreased renal blood flow and subsequent development of AKI in certain conditions that can cause hypovolemia (example: vomiting, diarrhea, poor oral intake, use of diuretics, gastrointestinal bleeding), use of non-steroidal anti-inflammatory agents, radiocontrast agents and progressive cirrhosis with decompensation and cirrhotic cardiomyopathy [13]. This ultimately leads to reduced effective arterial volume and activation of the renin-angiotensin-aldosterone system and sympathetic nervous system. Subsequently, there is afferent arteriolar constriction, further reduction in renal blood flow, and development of HRS-AKI [14].

Recently, a new theory has been proposed regarding the development of HRS. The development of HRS involves circulatory dysfunction as well as systemic inflammation. Bacterial translocation in cirrhotics lead to activation of bacterial products such as lipopolysaccharides, flagellin and bacterial DNA. Similarly, damaged hepatocytes release intracellular components including heat shock protein, double-stranded genomic DNA, adenosine triphosphate, etc. Their release leads to the release of pro-inflammatory cytokines like interleukin-6 (IL-6), tumor necrosis factor-a (TNF-a), interleukin 1b (IL-1b), etc., which are responsible for the development of renal impairment in patients with cirrhosis, acute on chronic liver failure and acute liver failure. 

Similarly, hepato-adrenal syndrome leads to a decrease in arterial pressure but an increase in renin and nor-epinephrine, nephropathy secondary to accumulation of intratubular bile casts, intra-abdominal hypertension in patients with refractory ascites have been implicated in the development of HRS [15].

Risk factors and prevention

Predictors of HRS include hyponatremia, high plasma renin activity, liver size, and severity of ascites. The prevalence of unprecipitated HRS-AKI is only 1.8% [16,17]. Large-volume paracentesis without albumin supplementation is the most common precipitating factor of HRS-AKI. Similarly, approximately 30% of patients with spontaneous bacterial peritonitis (SBP) develop HRS-AKI. [18] SBP associated HRS-AKI can potentially be prevented to a large extent by the use of intravenous albumin and antibiotics [19]. In patients with SBP, albumin is given at a dose of 1.5 g/kg on day 1 and 1 g/kg on day 3. In patients at risk of developing SBP, antibiotic prophylaxis prevents the development of SBP, reduces the risk of HRS-AKI and improves overall mortality [20].

Albumin infusion (6-8 grams per liter of fluid removed) post large-volume paracentesis (defined as paracentesis > 5 liters) significantly reduces the risk of development of HRS-AKI and improves the overall survival [21]. This protective effect is unique to albumin as compared to other volume expanders due to its antioxidant, anti-inflammatory, and endothelial stabilizing properties [22,23,24]. It also inactivates endotoxin [25]. Measures to prevent gastrointestinal bleed and other bacterial infections through appropriate screening and treatment should be done. Prevention of HRS is summarized in Table 3.

**Table 3:** Prevention of HRS AKI: acute kidney injury; AKD: acute kidney disease; CKD: chronic kidney disease; NSAIDs: nonsteroidal anti-inflammatory drugs.

Prevention of gastrointestinal bleeding and infections through appropriate screening and prophylaxis
Avoiding large-volume paracentesis without albumin
When renal dysfunction ensues in patients with cirrhosis, differentiation should be made whether it is AKI, CKD or AKD
Once AKI is diagnosed, a quick investigation should be done to determine the cause, and treatment of the precipitating factors should be done
Drugs such as vasodilators, NSAIDs, and beta-blockers should be discontinued
In patients with gastrointestinal bleed, packed red cell transfusion should be done to maintain hemoglobin > 7g/dl

Management of HRS

When a patient with liver disease has renal dysfunction, it should be differentiated whether it is AKI, CKD or AKD. Once AKI has been diagnosed, the cause should be investigated and management started immediately. Diuretics should be held. Beta-blockers should be stopped [26]. Precipitating factors for AKI including infection should be identified and treated. Drugs that can cause AKI such as vasodilators and non-steroidal anti-inflammatory agents should be discontinued. Volume replacement should be done as appropriate. Patients with diarrhea or excess diuresis may be treated with intravenous crystalloids. Patients with acute gastrointestinal bleeding should be treated with packed red cells to keep the hemoglobin above 7 g/dl [27].

If no obvious cause is found, treatment should be started with 20%-25% albumin at 1g/kg/day (with a maximum of 100 g of albumin per day) for two days. At the same time, intra-renal and post-renal etiologies should be ruled out with urine studies and renal ultrasonography. Acute tubular necrosis (ATN) should be distinguished from HRS-AKI. Determination of fractional excretion of sodium (FeNa) and urinary sodium is often unreliable in distinguishing ATN from HRS-AKI. However, Fractional excretion of urea (FeUrea) can be used to differentiate HRS-AKI from ATN in patients who take diuretics [28].

Since the definition of HRS-AKI does not rule out the possibility of kidney parenchymal damage, several urinary biomarkers reflecting tubular damage have been studied to differentiate ATN from HRS-AKI. Neutrophil gelatinase-associated lipocalin (NGAL), interleukin-18 (IL-18), kidney injury molecule-1 (KIM-1), and liver type fatty acid-binding protein (L-FABP) are few urinary biomarkers studied for this purpose. Compared to the patients without ATN, their value was found to be significantly higher in patients with ATN. This could be useful in differentiating causes of AKI in patients with cirrhosis [29].

The diagnosis of AKI-HRS should be considered if the above measures do not lead to improvement in renal function and specific therapy with vasoconstrictor and albumin should be initiated immediately. Early treatment of the HRS-AKI results in higher reversal rates and improved outcomes. Treatment of AKI-HRS includes vasoconstrictor in addition to albumin which should be initiated as soon as possible. Several randomized controlled trials have demonstrated that treatment with terlipressin plus albumin, led to significant improvement in renal function in patients with HRS type 1. The survival rate was better in patients who responded to treatment with terlipressin and albumin [30-32]. These results were, however, performed in patients with HRS type 1. ICA had revised and introduced the new criteria as a response to vasoconstrictor therapy is inversely related to the initial serum creatinine concentration. The new criteria do not have a cut-off serum creatinine value. This has given the advantage of making an early diagnosis so that the specific treatment can be started [10]. Further studies have to be done to evaluate the impact of the new definition on the effectiveness of pharmacologic treatments. 

Now, let us discuss the individual agents used in the management of HRS.

Vasoconstrictors

The use of vasoconstrictors leads to splanchnic vasoconstriction and reduces the portal pressure which in turn leads to an increase in effective arterial blood volume and renal blood flow. Renal perfusion increases with improvement in mean arterial pressure. This increase in mean arterial pressure is associated with a higher rate of HRS reversal [33].

The vasoconstrictors used are terlipressin, norepinephrine, and the combination of octreotide and midodrine which are all used in combination with albumin infusion. Terlipressin is a synthetic vasopressin analog with predominant vasopressin 1A receptor effects, mild vasopressin 1B receptor effect, and also has modest vasopressin 2 receptor effects. Acting on vasopressin 1A agonist, it constricts the splanchnic vessels [34]. Activation of vasopressin 1B receptor leads to the release of adrenocorticotrophic hormone and cortisol. This counteracts the relative adrenal insufficiency in cirrhotic patients [35]. However, its effect on vasopressin 2 receptors leads to retention of water and subsequent hyponatremia which is unwanted in patients in cirrhosis. Selepressin which is a selective vasopressin 1A receptor agonist has been investigated and already been studied to treat septic shock in animal models of sepsis. It has been shown to cause less hyponatremia, volume overload, and pulmonary edema in those models [36]. Its effect on human models and effectiveness on the treatment of HRS is yet to be studied. 

Terlipressin can be administered as both intravenous boluses and as a continuous infusion. Intravenous boluses can be given at 0.5 mg-1 mg every four to six hours to 2 mg every four hours. Continuous infusion can be given at 2 mg/day to 12 mg/day. Continuous infusion of octreotide has fewer side effects such as diarrhea, ischemia, and circulatory overload [37]. Thus, a careful clinical screening including an electrocardiogram is recommended before starting treatment. Treatment can be modified or discontinued according to the type and severity of side effects. The dose of terlipressin can be increased in a stepwise manner if the serum creatinine does not decrease by at least 25%. It should be used for a maximum of 14 days and stopped in case of a lack of response. It is called complete response if the final serum creatinine within 0.3 mg/dl from the baseline value. Partial response is defined as a regression of AKI but the final serum creatinine is ≥ 0.3 mg/dl from the baseline value. The response is achieved in about 40%-50% of patients. The recurrence rate is less than 20% and most of them respond to re-treatment [10].

Nor-epinephrine, which is given via central line at a dose of 0.5-3 mg/h is a systemic vasoconstrictor. It works through the activation of α-1 adrenergic receptors located on vascular smooth muscle cells. Its efficacy is similar to terlipressin with rates of HRS-AKI reversal ranging between 39%-70%, as shown in small randomized controlled trials [38-40]. It is started at a dose of 0.5mg/h to achieve an increase in mean arterial pressure of at least 10 mmHg or an increase in urine output of >200 ml in four hours. It can be increased by 0.5 mg/h to a maximum dose of 3 mg/h in case the parameters are not met. The patient has to be admitted to ICU for its administration via central line and though less expensive than terlipressin, the logistics can offset the cost-benefit. 

Midodrine is an oral α-1 agonist and has a mechanism of action similar to nor-epinephrine. Midodrine, when used alone does not improve renal function in patients with HRS [41]. Octreotide, which is a somatostatin analog, inhibits the secretion of glucagon, a splanchnic vasodilator. It also acts as a direct mesenteric vasoconstrictor [42]. Similar to midodrine, when used alone, octreotide has very limited benefit in patients with HRS as its activity is reduced by local nitric oxide release which is a vasodilator [43]. However, when used in combination, midodrine and octreotide has potential benefits and is the current standard of care in the United States for the treatment of HRS. The dose of midodrine is 5-15 mg three times a day. The dose can be increased by 2.5-5 mg every eight hours with the goal to increase the mean arterial pressure by at least 10-15 mmHg. Similarly, octreotide can be used either as 100-200 microgram given subcutaneously three times a day or as a continuous infusion of 50 micrograms/h. So far, only one study has been completed to compare the effect of terlipressin with midodrine and octreotide. The complete response rate for the terlipressin group was 55.5% compared to 4.8% with midodrine and octreotide. Similarly, the overall response rate for midodrine and octreotide was 28.6% compared to 70.4% with terlipressin [32].

The role of vasoconstrictors for the management of type 2 HRS or HRS-NAKI remains unclear as most of the studies have been done in HRS-AKI or type 1 HRS. 

Albumin

Infusion of albumin is the standard of care in the treatment of HRS. It should be used in combination with vasoconstrictors: Terlipressin, nor-epinephrine and midodrine, and octreotide. Albumin serves multiple roles in the management of HRS. It is a plasma volume expander and has positive cardiac inotropic effects [44]. The dose of albumin is 40-50 g/day and is continued for the duration of therapy along with vasoconstrictor drugs. The dose of albumin can be guided by serial measurement of central venous pressure can help to prevent circulatory overload [45].

Information about the treatment of HRS II or HRS-NAKI is very limited. Vasoconstrictive agents and albumin have been used in the treatment of HRS 2 and improvement in renal function has been observed as well. However, there is about a 50% recurrence rate after cessation of treatment. Trial of terlipressin may be helpful when selecting cirrhotic patients with renal failure as a bridge to transjugular intrahepatic portosystemic shunt or liver transplantation [46]. Since the patients with HRS 2 have refractory ascites resistant to diuretics and progressive renal failure, they are treated with repeated large-volume paracentesis or transjugular intrahepatic portosystemic shunt insertion [47].

The medical treatment of HRS is summarized in Table 4. 

**Table 4:** Medications used in the treatment of HRS. HRS: hepatorenal syndrome.

Terlipressin
Bolus dose: 0.5-1 mg every four to six hours up to 2 mg every four hours
Continuous infusion
Starting dose: 2 mg/day
Increment: Increase by 2 mg every 24-48 hours if the rate of decrease in serum creatinine is < 25% of the pretreatment value
Maximum dose: 12 mg/day
Nor-epinephrine (given as continuous infusion through central line)
Starting dose: 0.5 mg/h
Parameter to watch: Increase in mean arterial blood pressure by at least 10 mmHg or increase in urine output by 200 ml in four hours
Increment: 0.5 mg/h
Maximum dose: 3 mg/h
Midodrine
Starting dose: 5 mg by mouth every eight hours
Parameter to watch: Increase in mean arterial pressure by at least 10-15 mmHg
Increment: Increase the dose at eight-hour interval up to a maximum of 15 mg by mouth every eight hours
Octreotide
100-200 mcg given subcutaneously or continuous infusion of 50 mcg/h

Transjugular intrahepatic portosystemic shunt (TIPS)

The benefit of TIPS is mainly in patients with diuretics refractory ascites or patients who cannot tolerate diuretics and patients with uncontrolled variceal hemorrhage [48,49]. Its benefit in the management of HRS-AKI is still under investigation. Significant improvement in the renal function, marked improvement in portal pressure, and reduction in the activity of renin-angiotensin and sympathetic nervous system were seen in one small non-randomized study [50]. Similarly, a meta-analysis involving 128 patients showed significant improvement in serum creatinine, serum blood urea nitrogen, serum sodium, and urine output following TIPS both in HRS-1 and HRS-2 [51]. However, the study excluded patients with markedly elevated bilirubin, active infection, and hepatic encephalopathy, which means that TIPS could only be beneficial in a selected group of patients. 

Renal replacement therapy (RRT)

RRT can be used as a bridge to liver transplantation in patients with HRS-AKI who have volume overload, uremia, or metabolic derangements refractory to medical treatment. However, RRT alone does not improve survival [52]. The mortality rate in patients with cirrhosis and AKI who are ineligible for transplant is about 90% [53]. A limited trial of RRT can be considered in selected patients who are not liver transplant candidates depending upon the reversibility of other organ failures. However, a clear endpoint should be there as it is often futile.

Liver transplantation

Liver transplantation is the treatment of choice for patients with HRS-AKI. The functional nature of hepatic renal syndrome means that the kidney is expected to recover following successful liver transplantation. However, recovery of renal function is not universal and is dependent on multiple factors including age, co-morbid conditions like diabetes mellitus, unrecognized intrinsic kidney disease, and the most important being duration of kidney injury [54]. Serum creatinine after liver transplantation is higher in patients transplanted for HRS than for patients without HRS which has led to the question about the real nature of HRS [55]. The development of acute kidney injury before liver transplantation has been associated with a higher risk of chronic kidney disease, end-stage renal disease, and increased mortality post-transplantation [56,57]. Simultaneous liver-kidney transplant is recommended in such cases. In 2017 alone, 10% of all liver transplants were simultaneous liver-kidney transplantation in the United States [58].

Currently, in the United States, a listing policy now exists for simultaneous liver-kidney transplant based on prior consensus recommendations and includes the duration of AKI, need for dialysis, evidence of metabolic disease (hyperoxaluria, atypical hemolytic uremic syndrome, amyloidosis, and methylmalonic aciduria), and evidence of CKD [59].

Emerging therapy

Serelaxin is a recombinant human relaxin-2, which increases the renal blood flow by reducing renal vascular resistance and also reverses endothelial dysfunction [60]. A randomized phase II study done in compensated cirrhotic patients has shown an increase in renal blood flow by 65.4% from baseline with no effect on systemic blood pressure [61]. However, no studies have been done on patients with HRS. 

## Conclusions

HRS is a serious complication in patients with liver disease and has a poor prognosis. Improvements have been achieved in understanding the pathophysiology, diagnosis, and management of hepatorenal syndrome in recent years. In addition to the splanchnic and circulatory system changes, inflammation has been recognized as a major factor in the development of HRS. With more understanding, there is hope for earlier identification and introduction of new therapies to improve the outcome. However, the focus should be given to the prevention of HRS with timely treatment of infection and the use of albumin when needed. For patients diagnosed with HRS-AKI, terlipressin or nor-epinephrine plus albumin are the only effective treatment. Although, currently not approved for treatment in the United States, with promising data from different trials, it is hoped that Federal Drug Administration will approve terlipressin for clinical use in HRS. Liver transplant still remains the optimal treatment and the prospect of simultaneous liver-kidney transplantation is evolving. 

## References

[1] Russ KB, Stevens TM, Singal AK (2015). Acute kidney injury in patients with cirrhosis. J Clin Transl Hepatol.

[2] Ginès P, Solà E, Angeli P, Wong F, Nadim MK, Kamath PS (2018). Hepatorenal syndrome. Nat Rev Dis Primers.

[3] Arroyo V, Ginès P, Gerbes AL (1996). Definition and diagnostic criteria of refractory ascites and hepatorenal syndrome in cirrhosis. International Ascites Club. Hepatology.

[4] Caregaro L, Menon F, Angeli P (1994). Limitations of serum creatinine level and creatinine clearance as filtration markers in cirrhosis. Arch Intern Med.

[5] Belcher JM, Garcia-Tsao G, Sanyal AJ (2013). Association of AKI with mortality and complications in hospitalized patients with cirrhosis. Hepatology.

[6] Tsien CD, Rabie R, Wong F (2013). Acute kidney injury in decompensated cirrhosis. Gut.

[7] de Carvalho JR, Villela-Nogueira CA, Luiz RR (2012). Acute kidney injury network criteria as a predictor of hospital mortality in cirrhotic patients with ascites. J Clin Gastroenterol.

[8] Boyer TD, Sanyal AJ, Garcia-Tsao G (2011). Predictors of response to terlipressin plus albumin in hepatorenal syndrome (HRS) type 1: relationship of serum creatinine to hemodynamics. J Hepatol.

[9] Martín-Llahí M, Pépin MN, Guevara M (2008). Terlipressin and albumin vs albumin in patients with cirrhosis and hepatorenal syndrome: a randomized study. Gastroenterology.

[10] Angeli P, Ginès P, Wong F (2015). Diagnosis and management of acute kidney injury in patients with cirrhosis: revised consensus recommendations of the International Club of Ascites. J Hepatol.

[11] National Kidney Foundation (2002). K/DOQI clinical practice guidelines for chronic kidney disease: evaluation, classification, and stratification. Am J Kidney.

[12] Schrier RW, Arroyo V, Bernardi M, Epstein M, Henriksen JH, Rodés J (1988). Peripheral arterial vasodilation hypothesis: a proposal for the initiation of renal sodium and water retention in cirrhosis. Hepatology.

[13] Ruiz-del-Arbol L, Monescillo A, Arocena C (2005). Circulatory function and hepatorenal syndrome in cirrhosis. Hepatology.

[14] Facciorusso A, Amoruso A, Neve V, Antonino M, Prete VD, Barone M (2014). Role of vaptans in the management of hydroelectrolytic imbalance in liver cirrhosis. World J Hepatol.

[15] Simonetto DA, Gines P, Kamath PS (2020). Hepatorenal syndrome: pathophysiology, diagnosis, and management. BMJ.

[16] Ginès A, Escorsell A, Ginès P (1993). Incidence, predictive factors, and prognosis of the hepatorenal syndrome in cirrhosis with ascites. Gastroenterology.

[17] Wong F, Jepsen P, Watson H, Vilstrup H (2018). Un-precipitated acute kidney injury is uncommon among stable patients with cirrhosis and ascites. Liver.

[18] Follo A, Llovet JM, Navasa M (1994). Renal impairment after spontaneous bacterial peritonitis in cirrhosis: incidence, clinical course, predictive factors and prognosis. Hepatology.

[19] Sort P, Navasa M, Arroyo V (1999). Effect of intravenous albumin on renal impairment and mortality in patients with cirrhosis and spontaneous bacterial peritonitis. N Engl J Med.

[20] Fernández J, Navasa M, Planas R (2007). Primary prophylaxis of spontaneous bacterial peritonitis delays hepatorenal syndrome and improves survival in cirrhosis. Gastroenterology.

[21] Ginès P, Titó L, Arroyo V (1988). Randomized comparative study of therapeutic paracentesis with and without intravenous albumin in cirrhosis. Gastroenterology.

[22] Bernardi M, Caraceni P, Navickis RJ, Wilkes MM (2012). Albumin infusion in patients undergoing large-volume paracentesis: a meta-analysis of randomized trials. Hepatology.

[23] Fernández J, Monteagudo J, Bargallo X, Jiménez W, Bosch J, Arroyo V, Navasa M (2005). A randomized unblinded pilot study comparing albumin versus hydroxyethyl starch in spontaneous bacterial peritonitis. Hepatology.

[24] Zhang WJ, Frei B (2002). Albumin selectively inhibits TNF alpha-induced expression of vascular cell adhesion molecule-1 in human aortic endothelial cells. Cardiovasc Res.

[25] Chen TA, Tsao YC, Chen A (2009). Effect of intravenous albumin on endotoxin removal, cytokines, and nitric oxide production in patients with cirrhosis and spontaneous bacterial peritonitis. Scand J Gastroenterol.

[26] de Franchis R (2015). Expanding consensus in portal hypertension: Report of the Baveno VI Consensus Workshop: stratifying risk and individualizing care for portal hypertension. J Hepatol.

[27] Nadim MK, Durand F, Kellum JA (2016). Management of the critically ill patient with cirrhosis: A multidisciplinary perspective. J Hepatol.

[28] Carvounis CP, Nisar S, Guro-Razuman S (2002). Significance of the fractional excretion of urea in the differential diagnosis of acute renal failure. Kidney Int.

[29] Belcher JM, Sanyal AJ, Peixoto AJ (2014). Kidney biomarkers and differential diagnosis of patients with cirrhosis and acute kidney injury. Hepatology.

[30] Uriz J, Ginès P, Cárdenas A (2000). Terlipressin plus albumin infusion: an effective and safe therapy of hepatorenal syndrome. J Hepatol.

[31] Ortega R, Ginès P, Uriz J (2002). Terlipressin therapy with and without albumin for patients with hepatorenal syndrome: results of a prospective, nonrandomized study. Hepatology.

[32] Cavallin M, Kamath PS, Merli M (2015). Terlipressin plus albumin versus midodrine and octreotide plus albumin in the treatment of hepatorenal syndrome: A randomized trial. Hepatology.

[33] Velez JC, Nietert PJ (2011). Therapeutic response to vasoconstrictors in hepatorenal syndrome parallels increase in mean arterial pressure: a pooled analysis of clinical trials. Am J Kidney Dis.

[34] Jamil K, Pappas SC, Devarakonda KR (2017). In vitro binding and receptor-mediated activity of terlipressin at vasopressin receptors V1 and V2. J Exp Pharmacol.

[35] Tanoue A, Ito S, Honda K (2004). The vasopressin V1b receptor critically regulates hypothalamic-pituitary-adrenal axis activity under both stress and resting conditions. J Clin Invest.

[36] Laterre PF, Berry SM, Blemings A (2019). Effect of Selepressin vs Placebo on Ventilator- and Vasopressor-Free Days in Patients With Septic Shock: The SEPSIS-ACT Randomized Clinical Trial. JAMA.

[37] Cavallin M, Piano S, Romano A (2016). Terlipressin given by continuous intravenous infusion versus intravenous boluses in the treatment of hepatorenal syndrome: A randomized controlled study. Hepatology.

[38] Alessandria C, Ottobrelli A, Debernardi-Venon W (2007). Noradrenalin vs terlipressin in patients with hepatorenal syndrome: a prospective, randomized, unblinded, pilot study. J Hepatol.

[39] Sharma P, Kumar A, Shrama BC, Sarin SK (2008). An open label, pilot, randomized controlled trial of noradrenaline versus terlipressin in the treatment of type 1 hepatorenal syndrome and predictors of response. Am J Gastroenterol.

[40] Singh V, Ghosh S, Singh B (2012). Noradrenaline vs. terlipressin in the treatment of hepatorenal syndrome: a randomized study. J Hepatol.

[41] Angeli P, Volpin R, Piovan D (1998). Acute effects of the oral administration of midodrine, an alpha-adrenergic agonist, on renal hemodynamics and renal function in cirrhotic patients with ascites. Hepatology.

[42] Merkel C, Gatta A, Zuin R, Finucci GF, Nosadini R, Ruol A (1985). Effect of somatostatin on splanchnic hemodynamics in patients with liver cirrhosis and portal hypertension. Digestion.

[43] Pomier-Layrargues G, Paquin SC, Hassoun Z, Lafortune M, Tran A (2003). Octreotide in hepatorenal syndrome: a randomized, double-blind, placebo-controlled, crossover study. Hepatology.

[44] Bortoluzzi A, Ceolotto G, Gola E (2013). Positive cardiac inotropic effect of albumin infusion in rodents with cirrhosis and ascites: molecular mechanisms. Hepatology.

[45] (2018). EASL Clinical Practice Guidelines for the management of patients with decompensated cirrhosis. J Hepatol.

[46] Alessandria C, Venon WD, Marzano A, Barletti C, Fadda M, Rizzetto M (2002). Renal failure in cirrhotic patients: role of terlipressin in clinical approach to hepatorenal syndrome type 2. Eur J Gastroenterol Hepatol.

[47] Arroyo V, Fernández J (2011). Management of hepatorenal syndrome in patients with cirrhosis. Nat Rev Nephrol.

[48] Ginès P, Uriz J, Calahorra B (2002). Transjugular intrahepatic portosystemic shunting versus paracentesis plus albumin for refractory ascites in cirrhosis. Gastroenterology.

[49] Azoulay D, Castaing D, Majno P (2001). Salvage transjugular intrahepatic portosystemic shunt for uncontrolled variceal bleeding in patients with decompensated cirrhosis. J Hepatol.

[50] Guevara M, Ginès P, Bandi JC (1998). Transjugular intrahepatic portosystemic shunt in hepatorenal syndrome: effects on renal function and vasoactive systems. Hepatology.

[51] Song T, Rössle M, He F, Liu F, Guo X, Qi X (2018). Transjugular intrahepatic portosystemic shunt for hepatorenal syndrome: A systematic review and meta-analysis. Dig Liver Dis.

[52] Zhang Z, Maddukuri G, Jaipaul N, Cai CX (2015). Role of renal replacement therapy in patients with type 1 hepatorenal syndrome receiving combination treatment of vasoconstrictor plus albumin. J Crit Care.

[53] Fraley DS, Burr R, Bernardini J, Angus D, Kramer DJ, Johnson JP (1998). Impact of acute renal failure on mortality in end-stage liver disease with or without transplantation. Kidney Int.

[54] Israni AK, Xiong H, Liu J, Salkowski N, Trotter JF, Snyder JJ, Kasiske BL (2013). Predicting end-stage renal disease after liver transplant. Am J Transplant.

[55] Boyer TD, Sanyal AJ, Garcia-Tsao G (2011). Impact of liver transplantation on the survival of patients treated for hepatorenal syndrome type 1. Liver Transpl.

[56] Ojo AO, Held PJ, Port FK (2003). Chronic renal failure after transplantation of a nonrenal organ. N Engl J Med.

[57] Nadim MK, Genyk YS, Tokin C, Fieber J, Ananthapanyasut W, Ye W, Selby R (2012). Impact of the etiology of acute kidney injury on outcomes following liver transplantation: acute tubular necrosis versus hepatorenal syndrome. Liver Transpl.

[58] Formica RN, Aeder M, Boyle G, Kucheryavaya A, Stewart D, Hirose R, Mulligan D (2016). Simultaneous liver-kidney allocation policy: a proposal to optimize appropriate utilization of scarce resources. Am J Transplant.

[59] (2020). Organ Procurement and Transplantation Network. Policies. https://optn.transplant.hrsa.gov/media/1200/optn_policies.pdf..

[60] Conrad KP (2010). Unveiling the vasodilatory actions and mechanisms of relaxin. Hypertension.

[61] Snowdon VK, Lachlan NJ, Hoy AM (2017). Serelaxin as a potential treatment for renal dysfunction in cirrhosis: preclinical evaluation and results of a randomized phase 2 trial. PLoS Med.

